# *Leishmania mexicana* promastigotes inhibit macrophage IL-12 production via TLR-4 dependent COX-2, iNOS and arginase-1 expression

**DOI:** 10.1016/j.molimm.2011.05.013

**Published:** 2011-09

**Authors:** Muhannad Shweash, H. Adrienne McGachy, Juliane Schroeder, Thikryat Neamatallah, Clare E. Bryant, Owain Millington, Jeremy C. Mottram, James Alexander, Robin Plevin

**Affiliations:** a Division of Physiology & Pharmacology, Strathclyde Institute for Pharmacy & Biomedical Sciences, 27 Taylor Street, Glasgow G4 0NR, UK; b Infection, Immunity and Microbiology, Strathclyde Institute for Pharmacy & Biomedical Sciences, 27 Taylor Street, Glasgow G4 0NR, UK; c Department of Veterinary Medicine, University of Cambridge, Madingley Road, Cambridge CB3 0ES, UK; d Wellcome Trust Centre for Molecular Parasitology, Institute of Infection, Immunity and Inflammation, College of Medical, Veterinary and Life Sciences, University of Glasgow, Glasgow G12 8TA, UK

**Keywords:** *Leishmania mexicana*, Promastigotes, TLR-4, MAP kinase, Arginase-1, IL-12

## Abstract

The effects of *Leishmania mexicana* metacyclic promastigotes upon MAP kinase signalling in mouse bone marrow macrophages and subsequent expression of the disease regulatory proteins iNOS and COX-2 were studied. At a ratio of 5:1, promastigotes caused a marked increase in phosphorylation of the three major MAP kinases, ERK, p38 and JNK. MAP kinase signalling was substantially reduced in TLR-4^−/−^ but not TLR-2^−/−^ deficient macrophages and completely abolished in double TLR-2/4^−/−^ macrophages. A similar outcome was observed using cysteine peptidase B deficient amastigotes. Furthermore, whilst promastigotes had no independent effect on iNOS or COX-2 expression, they prolonged the induction of these proteins stimulated by LPS and enhanced PGE_2_ and NO production. Induction of COX-2 and iNOS was also TLR-4 dependent. Blockade of either PGE_2_ or NO production with indomethacin or l-NAME reversed promastigote inhibition of LPS induced IL-12 production. Promastigotes also increased macrophage arginase-1 expression and enhanced arginase activity, both of which were substantially reduced in TLR-4 but not TLR-2 deficient macrophages. Surprisingly, arginase inhibition by Nor-NOHA also caused a reversal of promastigote mediated inhibition of macrophage IL-12 production. These data demonstrate for the first time the role of TLR-4 in mediating the effects of *L. mexicana* promastigotes on MAP kinase activation, up-regulation of COX-2, iNOS as well as arginase-1 expression in macrophages and further shows that PGE_2_, NO and arginase activity all contribute substantially to the inhibition of host cell IL-12 production.

## Highlights

► TLR-4 activation by *Leishmania mexicana* promastigotes and CPB-deficient amastigotes. ► Prolonged, TLR-4 dependent iNOS and COX-2 expression by *L. mexicana* promastigotes. ► Enhanced TLR-4 dependent-arginase-1 expression. ► Regulation of IL-12 induction by an arginase-1 dependent mechanism.

## 1 Introduction

*Leishmania* are obligate intracellular parasites transmitted by sandflies as metacyclic promastigotes that infect primarily macrophages in the vertebrate host where they transform and multiply as amastigotes. Depending primarily on the species initiating infection, they cause a wide range of diseases such as cutaneous, mucocutaneous and visceral leishmaniasis in humans throughout the mediterranean, sub-tropical and tropical regions of the globe. Protective immunity is generally associated with an IL-12 driven type-1 response and IFN-γ production whilst non-healing disease is associated with a deficient type-1 response. Many studies now indicate an inability to produce or respond to IL-12 as the primary default mechanism leading to non-healing disease (reviewed by McMahon-Pratt and Alexander, 2004) and it is well established is that *Leishmania* promastigotes and amastigotes of all species studied to date are able to significantly inhibit the IL-12 production associated with host cell activation (reviewed by Kima, 2007).

The intracellular mechanisms through which *Leishmania* species regulate cellular responses, including IL-12 production, in macrophages and other host cells, remain unclear despite considerable study (Olivier et al., 2005). Nevertheless, a number of divergent effects are apparent, dependent upon parasite species, the life-cycle stage of the parasite (promastigote versus amastigote) and the cellular target. For example, host cell IL-10, generated via Fcγ receptor mediated uptake of opsonised parasites, has been shown to play a significant role in downregulating IL-12 production (Kane and Mosser, 2001; Thomas and Buxbaum, 2008). Furthermore, enhanced PGE_2_ production, mediated in part via parasite COX-2 induction (Matte et al., 2001; Reiner and Malemud, 1985) is also capable of inhibiting macrophage IL-12 although this has yet to be specifically demonstrated at the level of the infected macrophage. A similar inhibition of IL-12 action has also been demonstrated for nitric oxide (Huang et al., 1998), but few studies demonstrate if endogenous macrophage NO induced by the parasite infection is sufficient to promote this effect. Indeed, studies show inhibition of IL-12 production independently of these pathways (Weinheber et al., 1998); *Leishmania mexicana* and the related *Leishmania amazonensis* can directly mediate degradation of NFκB (Cameron et al., 2004) and JAK/STATs (Xin et al., 2008), key signalling pathways which regulate IL-12 transcriptional activity. These effects are associated with expression of parasite-derived cysteine peptidase B (CPB) (Abu-Dayyeh et al., 2010; Cameron et al., 2004) or surface molecules such as lipophosphoglycan (LPG) (Balaraman et al., 2005; Feng et al., 1999). However the mechanisms and pathways of regulation are not yet well defined.

Recently, a major role for toll-like receptors (TLRs) has been implicated in the actions of some *Leishmania* species. These studies indicate important roles for TLR-2 ligation and MyD88 in the control of infection with *Leishmania major* (de Veer et al., 2003; Kavoosi et al., 2010) and *Leishmania braziliensis* (Vargas-Inchaustegui et al., 2009), TLR-4 in control of *L. major* (Kropf et al., 2004a,b) and *Leishmania pifanoi* (Whitaker et al., 2008) and TLR-9 in controlling *Leishmania donovani*, *L. major* and *L. braziliensis* (Liese et al., 2007; Schneider et al., 2007). However, very few of these studies have assessed the cellular consequences of TLR engagement. Thus, whilst CPB deficient amastigotes (Cameron et al., 2004) as well as promastigotes (Lapara and Kelly, 2010; Ruhland and Kima, 2009) remain able to inhibit IL-12 production by activated host cells, it is possible that engagement of TLRs may be able to explain these effects (Vargas-Inchaustegui et al., 2009).

We show here that promastigotes activate MAP kinase signalling through a TLR-4 dependent mechanism to prolong COX-2 and iNOS expression and enhance PGE_2_ and NO production. This effect is mimicked by cysteine peptidase B deficient amastigotes. These mediators both function to subsequently down regulate IL-12 production. Furthermore, promastigotes enhance arginase-1 expression and activity again via a TLR-4 dependent pathway that also, unexpectedly, participates in negatively regulating IL-12 production. These data elucidate for the first time the multiple pathways simultaneously targeted by *L. mexicana* promastigotes and by implication amastigotes to inhibit production of IL-12.

## 2 Materials and methods

### 2.1 Medium and reagents

All cell culture reagents were from Invitrogen (Paisley, UK) and Cambrex BioScience (Veniers, Belgium). TC100 insect medium and LPS (from *Salmonella abortus*) were from Sigma (Poole, UK). Recombinant murine IL-4 was from BD Pharmingen (TM, USA). l-Arginine, Triton X-100, pepstatin A, aprotinin and antipain hydrochloride were from Calbiochem, UK. α-Isonitrosopropiophenone was obtained from Sigma (Poole, UK). MAP kinase and NF-κB (p65 isoform) antibodies were from Santa Cruz Biotechnology (CA, USA) and rabbit polyclonal anti-iNOS and anti COX-2 antibodies from Cayman Chemicals (Michigan, USA). Detection of arginase-1 used mouse monoclonal anti-arginase1 (BD, Drogheda, Ireland). The TLR-2, TLR-4 and TLR-2/4 deficient mice on a C57Bl6 background were obtained from Professor Akira S. Osaka University, Japan.

### 2.2 Parasites

*L. mexicana* (MNYC/BZ/62/M379) promastigotes were cultured in 25 cm^3^ culture flasks (IBS) in TC100 insect medium supplemented with 10% (v/v) FCS. The promastigotes were incubated at 26 °C for seven days, until the metacyclic stage was achieved. Amastigotes of cysteine peptidase B (*CPB*)-deficient mutants (Δ*cpb*) used in this study have been described previously (Cameron et al., 2004). Parasites were washed in RPMI at least three times before use.

### 2.3 Generation of bone marrow-derived macrophages (BMM) and infection

Bone marrow cells were obtained by flushing the femurs of C57BL/6 mice. Cells were cultured in DMEM, containing 10% (v/v) heat-inactivated FCS 30% (v/v) and L cell-conditioned medium. Once cells had become confluent after approximately 8–10 days, they were harvested by scraping into 5 ml of cold, sterile RPMI 1640 medium. The cell suspension was then washed three times; cells were diluted to the appropriate cell number using complete RPMI media and seeded in plates. These were then incubated at 33 °C/5% CO_2_ overnight to allow the cells to adhere to the plate. Cells were then stimulated, in a final volume of 500 μl, with LPS, IL-4 and/or with *L. mexicana* stationary phase metacyclic promastigotes harvested from *in vitro* cultures or lesion-derived CPB deficient amastigotes purified from infected mice (Cameron et al., 2004). A ratio of 5:1, parasite: macrophage was used except where indicated.

### 2.4 SDS-PAGE and Western blot analysis

Cells were exposed to vehicle or appropriate agonists for the relevant period of time. They were then washed twice with ice cold PBS before adding 200 μl of pre-heated Laemmli's sample buffer. The cells were then harvested with a rubber policeman and the chromosomal DNA sheared by repeatedly passing through syringe with a 21 gauge needle in sterile Eppendorf tubes. The tubes were boiled for 5 min to denature proteins and samples were stored at −20 °C until use. Proteins were separated on a 10% (for detection of MAPKs and arginase), 8.5% (for detection of COX-2) or 7.5% (for detection of iNOS) SDS-PAGE gel. The proteins separated by SDS-PAGE were transferred to nitrocellulose membranes by electrophoretic blotting following a standard protocol (Towbin et al., 1979). Proteins were identified as outlined previously using specific antibodies (Cameron et al., 2004).

### 2.5 Measurement of arginase activity

Murine BM-macrophage arginase activity was determined using an assay based on a reaction with α-isonitrosopropiophenon (ISPF), as previously described previously (Corraliza et al., 1994). Briefly, cells were harvested and lysed with 50 μl of 50 mM Tris–HCl buffer, pH 7.4, containing 0.1% Triton X-100, 5 μg/ml pepstatin A, 5 μg/ml aprotinin, 5 μg/ml antipain and MnCl_2_ 10 mM, pH 7.4. Arginine hydrolysis was performed by incubating the lysate with 25 μl of 0·5 M l-arginine (pH 9.7) at 37 °C for 60 min. The reaction was stopped by adding 400 μl of an acid solution containing H_2_SO_4_, H_3_PO_4_ and H_2_O in a ratio of 1:3:7 was added, along with 25 μl of a 9% solution of ISPF. The acid mixture and ISPF were also added to 100 μl aliquots of urea standards. Samples and standards were incubated at 95 °C for 45 min, and then allowed to cool for 10 min in darkness. Aliquots (200 μl) were added to wells of a 96 well plate and absorbance read at 540 nm on a Spectromax 190 plate reader. Arginase activity of the samples was calculated by comparison to a standard curve generated from known quantities of urea. One unit of arginase activity was defined as the enzyme activity that catalysed the production of 1 μMol urea/min.

### 2.6 NO release in infected BMM

The supernatant of cell culture medium was collected for NO analysis. Quantification of NO production, by measuring nitrite (a stable metabolite of NO) levels, was as previously described (Tsai et al., 1999). To 50 μl of well supernatant, 50 μl of Griess reagent (equal volumes of 2% (w/v) sulphanilamide in 5% (v/v) H_3_PO_4_ and 0.2% (w/v) naphylethylenediamine HCl in water) was added. After incubation for 10 min at room temperature in darkness, absorbance was read at 540 nm on a Spectromax 190 plate reader. Nitrite production was determined by comparison to a standard curve generated using known concentrations of NaNO_2_.

### 2.7 Macrophage cytokine detection

The concentration of IL-12 (p70/p40) present in cell culture supernatants was assayed by a two-site enzyme linked immunosorbant assay (ELISA) for murine rIL-12 (R&D Systems, Abingdon, UK). The concentration of PGE_2_ present in cell culture supernatant was measured by ELISA for Murine rPGE_2_ (R&D Systems, Abingdon, UK).

### 2.8 Statistical evaluation

Densitometry data generated from immunoblots was expressed as mean ± SEM for at least 3 separate experiments. The statistical significance of differences between mean values from control and treated groups were determined by the one-way analysis of variance (ANOVA) using GraphPad Prism^®^ Version 4.0 software or one tailed Student's Unpaired *t*-test. *p* < 0.05 was accepted as significant.

## 3 Results

### 3.1 Regulation of signalling pathways following *Leishmania mexicana* promastigote infection

**Fig. 1:**
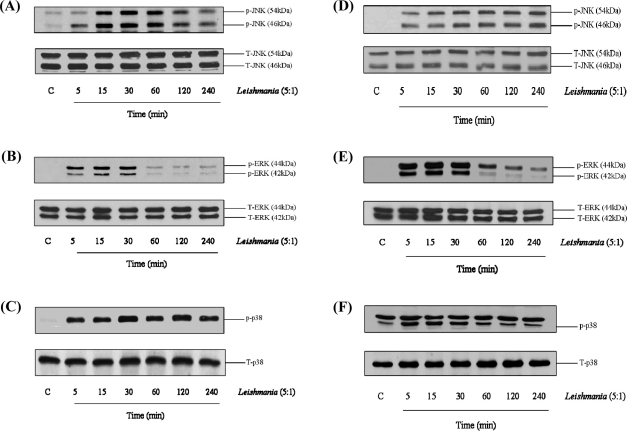
Promastigotes and Δ*cpb* amastigotes mediate increased MAP kinase signalling in macrophages. Cells (1 × 10^6^/well) were infected with *L.mexicana* promastigotes (A–C) or Δ*cpb* amastigotes (D-F) (ratio, 5:1) for the times indicated. Whole cell lysates were prepared, separated by SDS-PAGE, and then assessed for p-JNK1/2 and T-JNK (A and D), p-ERK1/2 and T-ERK (B and E) p-p38 MAPK and T-p38MAPK (C and F) as outlined in Section 2. The results are representative of 3 independent experiments with similar findings.

Having previously established a negative effect of *L. mexicana* amastigotes on kinase signalling pathways mediated by amastigote expressed CPB (Cameron et al., 2004) we tested the ability of promastigotes from the same species to modulate these pathways in mouse bone marrow derived macrophages. Alone, addition of promastigotes (5:1) resulted in a strong activation of MAP kinase signalling, with JNK activation apparent by 15 min after promastigote addition. ERK activation was more rapid but less sustained in comparison to JNK coming back to basal by 60 min, whilst p38 MAP kinase was rapidly induced and sustained for the full time course (Fig. 1A–C). Promastigotes alone also stimulated NFκB activation as assessed by cellular IκBα loss and the phosphorylation of p65 (Supplementary Fig. S1). However, pretreatment of mouse bone marrow derived macrophages with promastigotes had minor effect upon LPS induced signalling, and there was little consistent enhancement of MAP kinase phosphorylation (pERK, pp38 and pJNK) and NFκB activation (Supplementary Fig. S2).

We also sought to determine if cellular activation of MAP kinase was dependent upon either life cycle stage or the presence of the enzyme CPB which we have previously shown to degrade MAP kinases (Cameron et al., 2004). For this reason we used Δ*cpb* amastigotes at the same ratio (5:1). Addition of Δ*cpb* amastigotes to bone marrow derived macrophages also stimulated a similar rapid activation of the MAP kinase pathway, increasing the phosphorylation of JNK, ERK, p38 MAP kinase in a manner similar to promastigotes (Fig. 1D–F). Similar results were observed at the level of cellular IκBα loss and phosphorylation of p65 NFκB (not shown). Thus, *L. mexicana* infection of macrophages results in a marked increase in signalling events linked to innate immunity, but these changes are a component part of the parasite irrespective of the life cycle stage.

### 3.2 Requirement for TLR-4 in activation of macrophages by *L. mexicana* promastigotes

**Fig. 2:**
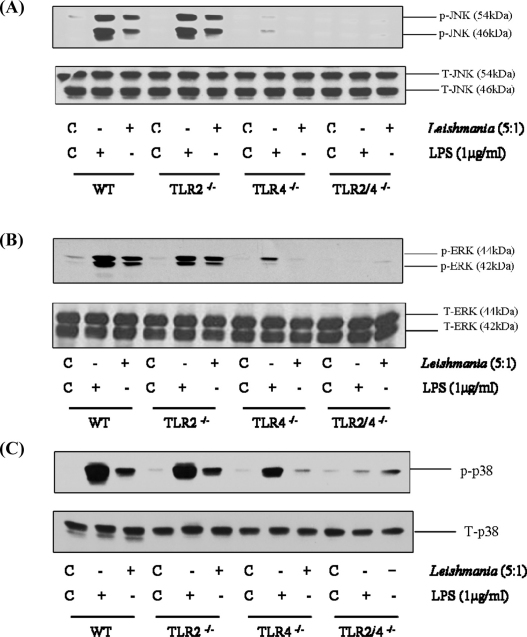
Promastigote activation of the MAP kinases is mediated by TLR-4. Macrophages derived from wild type (WT), TLR-2^−/−^, TLR-4^−/−^ or TLR-2/4^−/−^ mice (1 × 10^6^/well) were infected with *L. mexicana* promastigotes (ratio, 5:1) or LPS for the times indicated. Whole cell lysates were prepared, separated by SDS-PAGE, and then assessed for (A) p-JNK1/2 (46/54 kDa) and T-JNK, (B) p-ERK1/2 (44/42 kDa) and T-ERK (C) p-p38 MAP kinase and T-p38MAPK. The results are representative of 3 independent experiments with similar findings.

**Fig. 3:**
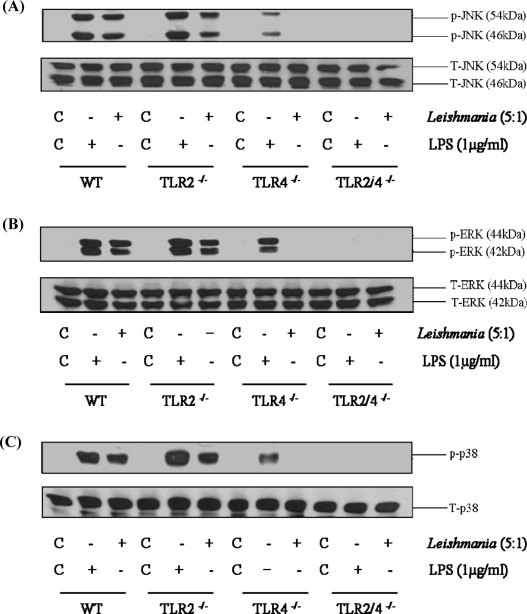
TLR-4 mediates Δ*cpb* amastigote activation of MAP kinases. Macrophages derived from wild type, TLR-2^−/−^, TLR-4^−/−^ or TLR-2/4^−/−^ mice (1 × 10^6^/well) were infected with *L.mexicana* Δ*cpb* amastigotes (ratio, 5:1) for the times indicated. Whole cell lysates were prepared, separated by SDS-PAGE, and then assessed for (A) p-JNK1/2 (46/54 kDa) and T-JNK (B) p-ERK1/2 (44/42 kDa) and T-ERK (C) p-p38 MAPK and T-p38 MAPK. The results are representative of 3 independent experiments with similar findings.

Recent studies have shown the potential for *L. major* to activate TLR-2 (de Veer et al., 2003; Kavoosi et al., 2010) and TLR-4 (Kropf et al., 2004a,b). We therefore compared MAP kinase signalling following *L. mexicana* promastigote infection in macrophages deficient in TLR-2 and TLR-4 or both (Fig. 2). In macrophages from wild type mice, *L. mexicana* promastigotes stimulated an increase in phosphorylation of all three MAP kinases, JNK (Panel A), ERK (Panel B) and p38 MAP kinase (Panel C), confirming our previous observations. Activation of MAP kinase signalling was only slightly reduced in TLR-2^−/−^ macrophages. However, in TLR-4^−/−^ macrophages, phosphorylation of all three MAP kinases were markedly affected, ERK and JNK phosphorylation was essentially abolished whilst p38 MAP kinase was significantly reduced. This residual activation of p38 MAP kinase is unclear but could be a consequence of entry of the pathogen, an effect which we have noted previously for *Salmonella* (Royle et al., 2003). A similar pattern of sensitivity was observed for LPS induced kinase signalling, confirming the predicted pattern of sensitivity for a known TLR-4 ligand. We did however observe a residual effect in TLR-4^−/−^ macrophages particularly in response to LPS, this is likely due to contaminating fragments which are able to activate TLR-2. Nevertheless, in TLR-2/4^−/−^ macrophages, MAP kinase signalling in response to *L. mexicana* was completely abolished. A similar TLR-4 dependency was observed for Δ*cpb* amastigote stimulation of MAP kinase signalling (Fig. 3) suggesting a stage-independent activation. Thus, activation of macrophages by *L. mexicana* promastigotes is largely through a TLR-4 dependent pathway, with a neglible role for TLR-2 signalling.

### 3.3 *Leishmania mexicana* promastigotes enhance COX-2 and iNOS expression in a TLR-4 dependent manner

**Fig. 4:**
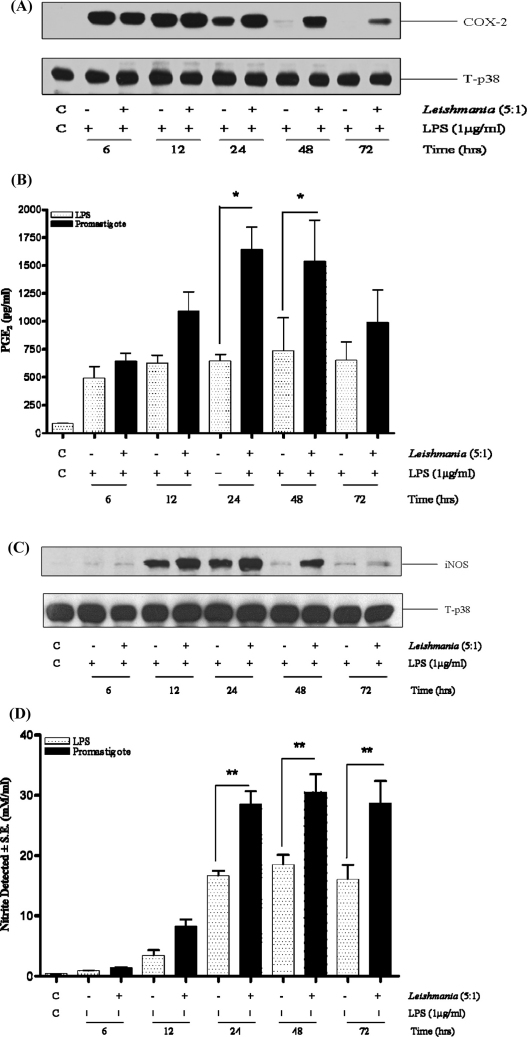
Promastigotes prolong LPS induced COX-2 and iNOS expression. Cells (1 × 10^6^/well) were infected with *L. mexicana* promastigotes (ratio, 5:1) for 2 h and then stimulated with LPS (1 μg/ml) for the times indicated. Whole cell lysates were prepared, separated by SDS-PAGE, and then assessed for COX-2 (Panel A) or iNOS (Panel C) as outlined in Section 2. In Panels B and D, supernatants were collected and assessed for PGE_2_ or nitrate accumulation respectively. Each value represents the mean ± S.E.M. The results are representative of at least 3 independent experiments with similar findings. * and ** indicates *p* < 0.05 and 0.01 compared to LPS alone.

We next examined functional expression of COX-2 and iNOS, two downstream events linked to TLR signalling (Fig. 4). Infection with promastigotes alone did not induce macrophage COX-2 or iNOS protein expression at any time point (data not shown). However, infection of macrophages with *L. mexicana* promastigotes prolonged the kinetics of protein expression induced by LPS such that after 24 h when LPS induced responses waned, COX-2 and iNOS expression was maintained for a further 48 h by the presence of promastigotes (Fig. 4A and C). Concomitant to increased COX-2 and iNOS expression, PGE_2_ and NO release were similarly enhanced by promastigote infection particularly at the 24 and 48 h time points (Fig. 4B and D). Control experiments indicate that this was not dependent upon enhanced activation of IRF-3 phosphorylation known to be a key component of TLR-4 induction of iNOS. Whilst LPS enhanced IRF-3 phosphorylation this was not further modified by the presence of promastigotes (Supplementary Fig. S3).

**Fig. 5:**
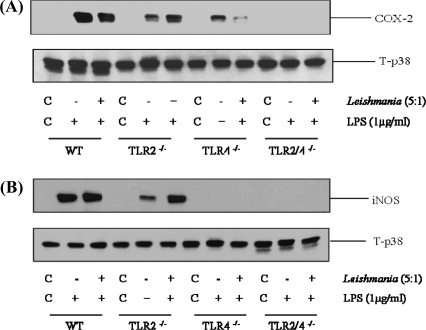
TLR-4 mediates sustained iNOS and COX-2 in response to promastigotes. Macrophages derived from wild type (WT), TLR-2^−/−^, TLR-4^−/−^ or TLR-2/4^−/−^ mice (1 × 10^6^/well) were infected with *L.mexicana* promastigotes (ratio, 5:1) for 2 h and then stimulated with LPS (1 μg/ml) for 24 h. Whole cell lysates were prepared, separated by SDS-PAGE, and then assessed for COX-2 (Panel A) or iNOS (Panel B) and T-p38 MAPK as outlined in Section 2. The results are representative of at least 3 independent experiments with similar findings.

Nevertheless, the enhancing effect of promastigotes on LPS induced macrophage COX-2 and iNOS expression were again dependent upon TLR-4 activation (Fig. 5). In TLR-2^−/−^ macrophages, the reduction in signal was marginal, however in TLR-4^−/−^ macrophages expression of COX-2 was substantially although not completely reduced. In addition, iNOS expression was essentially abolished in TLR-4^−/−^ deficient macrophages. In macrophages derived from TLR-2/4^−/−^ mice, both COX-2 and iNOS signals were abolished. A similar result was obtained for Δ*cpb* amastigotes (results not shown). Thus early activation of MAP kinase signalling through TLR-4 reflects a similar receptor dependency at the level of downstream iNOS and COX-2 expression.

### 3.4 *L. mexicana* promastigote inhibition of macrophage IL-12 production is a result of prolonged PGE_2_ and NO induction

**Fig. 6:**
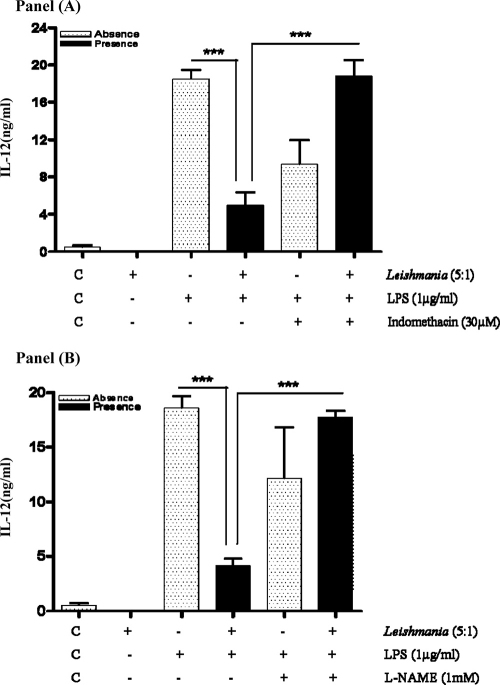
Prevention of PGE_2_ and NO formation reverses promastigote inhibition of IL-12 production. Cells (1 × 10^6^/well) were infected with *L.mexicana* promastigotes (ratio, 5:1) for 2 h and then stimulated with LPS (1 μg/ml) for 6 h. Indomethacin (30 μM) or l-NAME (1 mM) was then added and cells incubated for a further 18 h. Samples were assessed for IL-12 production as outlined in Section 2. Each value represents the mean ± S.E.M. of at least 3 experiments performed in triplicate, *** indicates *p* < 0.05 compared to LPS alone.

In order to determine the functional consequences of prolonged macrophage NO and PGE_2_ formation induced by promastigotes through TLR-4, we examined the inter-relationship with IL-12 production. We have previously demonstrated that amastigotes inhibit IL-12 in significant part due to CPB mediated degradation of the NFκB pathway (Cameron et al., 2004). Pre-infection of macrophages with promastigotes, which lack the high levels of CPB found in amastigotes, prior to LPS treatment also abolished IL-12 production (Fig. 6, Panels A and B). Inhibition proved to be cytokine specific as LPS induction of TNFα, IL-1β, IL-6 or IL-10 was not affected (Supplementary Fig. S4). However, we also found that addition of the COX-2 inhibitor indomethacin at 6 h subsequent to LPS, substantially reversed the effect of promastigotes on macrophage IL-12 production after 24 h (Panel A). Interestingly, pretreatment with a non-selective NOS inhibitor l-NAME which abolished NO release (not shown) also reversed the inhibitory effect of promastigotes on macrophage IL-12 production (Panel B).

### 3.5 *L. mexicana* promastigotes induce macrophage arginase-1 expression and activity which also downregulates IL-12 production

**Fig. 7:**
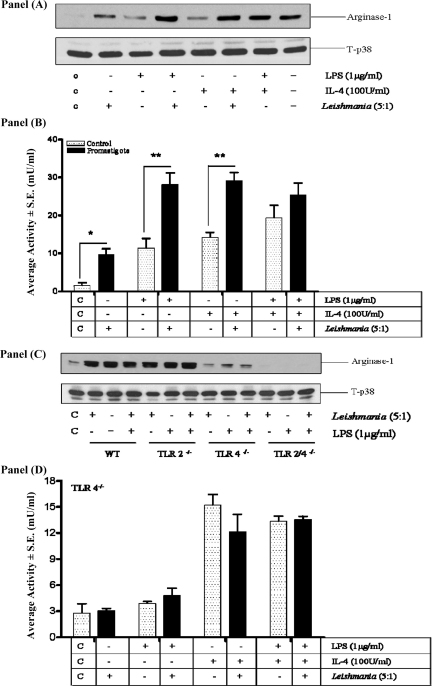
Promastigotes increase arginase-1 expression and activity by a TLR-4 dependent mechanism. Cells (1 × 10^6^/well) from WT (Panels A–C), or TLR-4^−/−^ mice (Panels C and D) were infected with *L.mexicana* promastigotes (ratio, 5:1) for 2 h and then stimulated with LPS or IL-4 for 24 h. Arg-1 expression (Panels A and C) or activity (Panels B and D) was measured as outlined in Section 2. The results are representative of 3-independent experiments with similar findings. Where indicated each value represents the mean ± S.E.M. *, ** indicates *p* < 0.05 or 0.01 compared to LPS alone.

We also examined whether TLRs 2 and 4 were involved in other pathways which may mediate the actions of promastigotes upon macrophage function (Fig. 7). Consistently we found that promastigotes alone were sufficient to induce arginase-1 expression and activity, as well as enhancing LPS or IL-4-induced arginase-1 (Panels A and B). By contrast, induction of arginase-1 by promastigotes was substantially reduced in TLR-4^−/−^ macrophages but not in macrophages derived from TLR-2^−/−^ mice (Panel C). In TLR-2/4^−/−^ macrophages arginase-1 levels were reduced even further to below the control base line. This was also reflected at the level of arginase activity (Panel D). Neither promastigotes nor LPS induced measurable macrophage arginase activity in the absence of TLR-4 and no additive effects were observed between these agents. Furthermore, whilst IL-4 was still able to induce arginase, there was no additive effect with either promastigotes alone or in combination with LPS (Panel D).

**Fig. 8:**
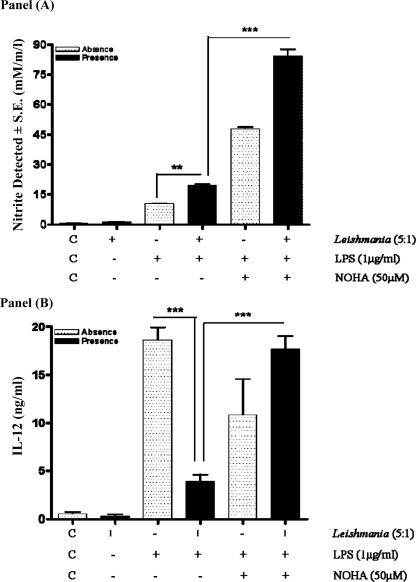
Arginase blockade reverses promastigote mediated inhibition of IL-12 production. Cells (1 × 10^6^/well) were infected with *L. mexicana* promastigotes (ratio, 5:1) for 2 h, then stimulated with LPS (1 μg/ml) for 6 h prior to the addition of nor-NOHA (50 μM) for a further 18 h. Samples were assessed for IL-12 production as outlined in Section 2. Each value represents the mean ± S.E.M. of at least 3 experiments performed in triplicate. *** indicates *p* < 0.05 compared to LPS alone.

Finally, we sought to determine if arginase induction mediated by promastigote infection could regulate the interplay between NO and IL-12 production (Fig. 8). Macrophages were pre-infected with promastigotes and then stimulated with LPS prior to addition of the arginase inhibitor, nor-NOHA (50 μM). When cells were treated with the arginase inhibitor, this substantially increased the release of NO in response to LPS in combination with promastigotes, indicating that arginase activity limits the levels of NO within the macrophage. Surprisingly, the intervention at this concentration which enhanced NO release, also reversed the inhibitory effect of promastigotes on IL-12 production (Panel B). Similar results were obtained with Δ*cpb* amastigotes (results not shown).

## 4 Discussion

In order to successfully parasitize the vertebrate host *Leishmania* species, such as *L. mexicana* (Rodriguez-Sosa et al., 2001), must subvert the innate inflammatory response of host cells that results in the IL-12 production that is pivotal in initiating the development of type-1 protective immunity. Our present studies highlight novel and multiple mechanisms utilized by *L. mexicana* promastigotes as well as amastigotes to modulate host cell intracellular signalling functions to achieve this objective. We find that promastigotes and also CBP deficient null mutant amastigotes, by utilizing a TLR-4 dependent mechanism, mediate the phosphorylation of ERK, p38 and JNK to enhance LPS induced macrophage iNOS and COX-2 expression and in addition, enhance arginase-1 expression in naïve as well as activated host cells. Furthermore, we clearly demonstrate using specific inhibitors that the activities associated with all three enzymes down regulate macrophage IL-12 production. Overall our data sheds significant new light on how the “mexicana complex” parasites modulate host cell function to subvert the induction of protective immunity.

Our initial experiments demonstrated a pattern of kinase activation in response to promastigotes which mimicked closely responses following TLR engagement and reflected some of the characteristics of kinase activation demonstrated by some other *Leishmania* species (Ben-Othman et al., 2008). Our experiments, however, revealed precisely the TLR, which mediates the effects of promastigotes on MAP kinase signalling, namely TLR-4. TLR-4 is linked to both the MAP kinase pathway and NFκB through the activation of MyD88 signalling, a model consistent with the profile of kinase activation recorded in this paper (O’Neill et al., 2009). Recent studies have implicated a role for TLR-dependent events in the actions of *Leishmania* species including TLR-2 (Becker et al., 2003), TLR-4 (Kropf et al., 2004a) and MyD88 (Vargas-Inchaustegui et al., 2009). Although there is evidence that TLR-2 may play a regulatory role during infection with *L. braziliensis* (Vargas-Inchaustegui et al., 2009) and downregulate DC IL-12 production, all other studies implicate TLRs and TLR-4 ligation and MyD88 signalling in particular in the generation of protection against infection. Interestingly, we also demonstrate activation of MAP kinase in response to infection with Δ*cpb* amastigotes, again through a TLR-4 dependent mechanism. This suggests that irrespective of the life cycle stage, *L. mexicana* has the ability to engage with a pathogen recognition receptor. For amastigotes this is significant if we also consider that this stage, which normally express the enzyme CPB, has as a primary mechanism of action, the degradation of intracellular signalling proteins such as NFκB intermediates and the MAP kinases (Cameron et al., 2004). Thus, we have revealed that amastigotes can regulate MAP kinase signalling in two ways, one positively through TLR-4 engagement, the other negatively via degradation of intracellular kinases.

We also observed effects of promastigotes upon macrophage iNOS and COX-2 expression downstream of MAP kinase signalling. Although infection with promastigotes alone was not sufficient to induce either protein, it resulted in a prolonged induction of both COX-2 and iNOS in response to LPS stimulation and enhanced macrophage PGE_2_ and NO release over an extended period. This effect was dependent upon TLR-4 activation, the first time regulation of COX-2 and iNOS has been described for *L. mexicana* promastigotes via this receptor. It was surprising that promastigotes were unable to increase iNOS and COX-2 *per se*, since TLR-4 ligation is sufficient to do this (O’Neill et al., 2009), however lower levels of receptor occupancy may be a factor. Also it is possible that promastigotes may have differential effects on other pathways required for iNOS and COX-2 production such as NFκB and IRF-3 (O’Neill et al., 2009). Indeed our studies have shown promastigotes do not enhance IRF3 phosphorylation, a key event in iNOS expression induced in response to LPS (O’Neill et al., 2009). Thus some other mechanism must be responsible for the effects observed in this paper.

Enhancing PGE_2_ release through prolonged COX-2 expression would be an outcome consistent with studies showing PGE_2_ to be a virulence factor (Guimaraes et al., 2006); exogenous PGE_2_ increases *L. amazonensis* parasite load in BALB/c macrophages (Pinheiro et al., 2009) and indomethacin treatment *in vitro* enhances IL-12 and IFN-γ production from splenocytes derived from *L. mexicana* infected BALB/c mice (Perez-Santos and Talamas-Rohana, 2001). Indeed prevention of PGE_2_ release by treatment with indomethacin subsequently reversed the inhibition of macrophage IL-12 production by promastigotes indicating that enhanced COX-2 expression is functionally relevant in the actions of *L. mexicana*. Although a number of previous studies demonstrate that exogenous PGE_2_ can reduced macrophage IL-12 production (Iwasaki et al., 2003; Monteleone et al., 1999), few focus on endogenous production following *Leishmania* infection and this is one of the first studies which relates sustained PGE_2_ production induced by any *Leishmania* species to an effect upon IL-12 production within the same macrophage. This may be related to the fact that *Leishmania* infection *per se* may not be sufficient to induce COX-2 expression and an additional stimulus is required (Farrell and Kirkpatrick, 1987). In mammary carcinomas, production of PGE_2_ is markedly increased resulting in inhibition of endogenous IL-12, an effect which can be reversed by non-selective blockade of COX activity (Mitsuhashi et al., 2004).

Prevention of NO production also significantly reversed the inhibition of IL-12 by promastigotes. This is consistent with studies which show that pharmacological or genetic modulation of NO levels can regulate IL-12 production (Boddupalli et al., 2007; Huang et al., 1998; Xiong et al., 2004). Although increase in NO levels is usually associated with parasite killing the fact that in this present study promastigotes enhanced NO formation would appear to be paradoxical. However, studies show that promastigotes (Gomes et al., 2003) and amastigotes (Mukbel et al., 2007) from *L. amazonensis*, a “mexicana complex” parasite, are more resistant to NO than *L. major*. Recent evidence indicates that, unlike *L. major*, there is in fact enhanced replication of the amastigote stage of *L. amazonensis* in IFN-γ-stimulated murine macrophages despite increased NO production (Wanasen et al., 2007). The evidence would suggest that macrophage killing of *L. amazonensis* unlike *L. major* requires NO and additionally, superoxide (Mukbel et al., 2007). Consequently a certain level of NO induction by *L. mexicana* complex parasites need not be detrimental to the parasite but in fact could actually promote infection by inhibiting IL-12 production and the development of a protective type-1 response.

Induction of arginase by *L. amazonensis* has also been shown to enhance replication of the amastigote state of the parasite (Qi et al., 2004; Wanasen et al., 2007) whilst arginase activity is also associated with susceptibility to *L. major* infection in BALB/c mice (Kropf et al., 2005). Our studies also revealed the potential for *L. mexicana* promastigotes to be involved in the regulation of arginase-1 expression in the macrophage through TLR-4. This finding is different from a study which demonstrates that in TLR-4 deficient mice arginase activity in response to *L. major* is enhanced, implicating that TLR-4 negatively controls arginase-1 induction (Kropf et al., 2004a). Other studies, whilst assessing TLR involvement in *Leishmania* host-cell function have not examined coupling to arginase-1 expression. Recently TLR-2 has been implicated in arginase expression and pathogenicity in response to *Mycobacterium tuberculosis* or *Toxoplasma gondii* (El Kasmi et al., 2008) with protective immunity being thwarted by reduced NO production. However, a recent study in bone marrow derived macrophages has demonstrated TLR-4 ligation via LPS to enhance both *nos-2* and *arg-1* expression and NO and arginase activities (Menzies et al., 2010) which would be consistent with *L. mexicana* promastigotes utilizing TLR-4 as shown in this present study. Consequently, our results reveal crucial differences in the utilization of TLRs in regulating arginase in response to different pathogens. We also elucidated the involvement of arginase in the cellular responses to promastigotes, in particular regulation of IL-12 production. This intriguing result is consistent with a recent study that has linked arginase expression to inhibition of IL-12 production during *Schistosoma mansoni* infection (Herbert et al., 2010). Nevertheless, consistent with arginase and iNOS competing for the same substrate l-arginine inhibition enhanced NO production which should function to reduce IL-12, but surprisingly, did not. This paradox suggests potential differences in the ability of each intermediate to negatively regulate IL-12 production; arginase being implicated as more potent/efficacious than NO. Overall our data would suggest that *L. mexicana* promastigotes and CPB deficient amastigotes, through TLR-4, are able to prolong and enhance PGE_2_, NO and arginase production all of which limit macrophage IL-12 production and subvert the induction of type-1 protective immune responses. These data elucidate for the first time the multiple pathways simultaneously targeted by *L. mexicana* promastigotes to inhibit production of IL-12.

## Conflict of interest

The authors declare no conflict of interests from this work.

## Glossary

COX-2: cyclooxygenase-2
ERK: extracellular regulated kinase
iNOS: inducible nitric oxide synthase
IL: interleukin
JNK: c-jun N-terminal kinase
MAP kinase: mitogen-activated protein kinase
NO: nitric oxide
PGE_2_: prostaglandin E_2_
Nor-NOHA: N^ω^-hydroxy-nor-arginine
M.O.I.: multiplicity of infection
TLR-4: toll-like receptor-4.
