# Supplementary material for: Leishmania mexicana promastigotes inhibit macrophage IL-12 production via TLR-4 dependent COX-2, iNOS and arginase-1 expression
Source: Mol Immunol. 2011 Sep;48(15-16):1800–8. doi: 10.1016/j.molimm.2011.05.013 (PMC3173610; doi:10.1016/j.molimm.2011.05.013)
Supplement: Supplementary file 1 [file mmc1.doc]

**Figure S 1: *Leishmania* promastigotes induce IκB-α loss and p65 phosphorylation**

Macrophages (1x10^6^) were infected with *L. mexicana* promastigotes (ratio 5:1) for up to 4h. At the time points indicated, cells were lysed in protein sample buffer, separated on SDS-Gels and analysed for IκB-α and p65 phosphorylation (p-p65). Detection of total p65 (T-p65) was used as loading control.

We examined the potential of *L. mexicana* promastigotes to interfere with the NFκB pathway of host macrophages. Parasite induced IκB-α degradation can be found as early as 30 min after infection and is maintained throughout the duration of the experiment (Figure S1). This coincides with phosporylation of p65, which is still detected at 4 hours after infection with *L. mexicana*. However, the loss of IκB-α is only partial.
