# Supplementary material for: Leishmania mexicana promastigotes inhibit macrophage IL-12 production via TLR-4 dependent COX-2, iNOS and arginase-1 expression
Source: Mol Immunol. 2011 Sep;48(15-16):1800–8. doi: 10.1016/j.molimm.2011.05.013 (PMC3173610; doi:10.1016/j.molimm.2011.05.013)
Supplement: Supplementary file 2 [file mmc2.doc]

A)

C)

B)

D)

**Figure S 2: Effects of *L. mexicana* promastigotes on host macrophage MAPK and NFκB pathways**

Macrophages (1x10^6^) were infected with *L. mexicana* promastigotes (ratio 5:1) or left uninfected for 2h after which cells were treated with 1μg/ml LPS for the times indicated. Whole cell lysates were prepared, separated via SDS-PAGE and analysed for phosphorylation of JNK (A), ERK (B), p38 (C) and p65 (D) as well as their respective total protein levels (A-D) and IκB-α loss (D). The results are representative of 3 independent experiments with similar findings.

This experiment was performed to examine the effects of *L. mexicana* promastigotes on LPS induced activation of MAPK and NFKB pathways in host macrophages. While JNK remains unaffected (Figure S 2A), a slight decrease in ERK phosphorylation was observed at early time points (15 to 30 min) in cells infected with *L. mexicana* (Figure S 2B). A similar , small effect was seen for p38, although at later time points (60 to 120 min) this effect was veered towards higher p38 phosphorylation in *Leishmania*-infected macrophages (Figure S 2C). The parasite appears to be able to enhance the NFκB pathway, as an increase in IκB-α loss and p65 phosphorylation was observed after 30 min on LPS stimulation. This shows that *L. mexicana* promastigotes are able to interfere with LPS induced activation of host MAPK and NFκB pathways but only to a very minor extent.
