# Supplementary material for: Leishmania mexicana promastigotes inhibit macrophage IL-12 production via TLR-4 dependent COX-2, iNOS and arginase-1 expression
Source: Mol Immunol. 2011 Sep;48(15-16):1800–8. doi: 10.1016/j.molimm.2011.05.013 (PMC3173610; doi:10.1016/j.molimm.2011.05.013)
Supplement: Supplementary file 3 [file mmc3.doc]

**Figure S 3: Promastigotes show no effect on IRF-3 pathway**

Macrophages (1x10^6^) were stimulated with LPS (100 ng/ml, lane 1), infected with *L. mexicana* promastigotes alone (ratio 5:1, lane 2) or infected with *L. mexicana* promastigotes for 30 min and subsequently stimulated with LPS (lane 3). After 8h whole cell lysates were prepared, separated by SDS-PAGE and assessed for phosphorylated IRF-3 (p-IRF-3) and β-Tubulin as respective loading control.

We examined whether the IRF-3 pathway mediates the action of *L. mexicana* promastigotes on macrophage responses. We found that LPS (100 ng/ml) alone caused an activation of the IRF-3 pathway. Phosphorylation of IRF-3 was increased as early as 30 min, giving a maximum response at 8 hours (Figure S 3) before returning to a basal line after 24 hours. Promastigotes alone (ratio 5:1) failed to activate the IRF-3 pathway and did not alter LPS induced IRF-3 phosphorylation suggesting that promastigotes have no effect on the IRF-3 pathway that is required for iNOS and COX-2 production.
