# Supplementary material for: Leishmania mexicana promastigotes inhibit macrophage IL-12 production via TLR-4 dependent COX-2, iNOS and arginase-1 expression
Source: Mol Immunol. 2011 Sep;48(15-16):1800–8. doi: 10.1016/j.molimm.2011.05.013 (PMC3173610; doi:10.1016/j.molimm.2011.05.013)
Supplement: Supplementary file 4 [file mmc4.doc]

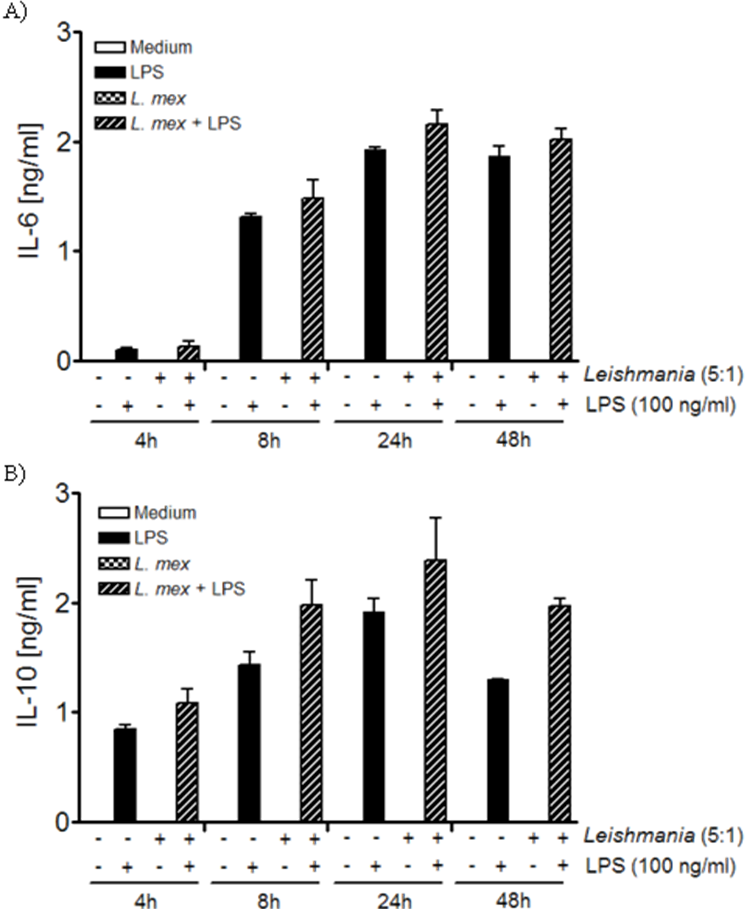


**Figure S 4: Effect of *Leishmania* promastigotes on macrophage cytokine production**

Macrophages (2x10^5^) were infected with *L. mexicana* promastigotes (ratio 5:1) or left untreated (Medium and LPS control) for 1h at 33°C before unattached parasites were removed, cells washed with PBS and fresh medium supplemented with or without 100 ng/ml LPS was added. For the time points indicated cell free supernatant was analysed for cytokine production by ELISA. Each treatment was done in triplicates and error bars represent standard deviation (SD).

We examined the effect of *L. mexicana* promastigotes on macrophage cytokine production (IL-6, IL-10, TNFα and IL-1β) per se and after activation with LPS (100 ng/ml). While cytokine levels for TNFα and IL-1β did not increase above background (data not shown), IL-6 and IL-10 were both only detected after activation of macrophages with LPS (Figure S 4). *L. mexicana* promastigotes on their own did not seem to be able to induce these cytokines nor did they alter LPS induced IL-6. For IL-10 however, a slight increase or prolongation was detected after 48h.
